# Validation of an UHPLC-MS/MS Method for Simultaneous Analysis of 11 Mycotoxins in Wheat Flour Using Immunoaffinity Column

**DOI:** 10.22037/ijpr.2019.112398.13735

**Published:** 2019

**Authors:** Ghasem Heidari, Seyed Jamal Hashemi Hazaveh, Bahram Daraei, Mansour Bayat

**Affiliations:** a *Department of Pathobiology, Science and Research Branch, Islamic Azad University, Tehran, Iran. *; b *Department of Medical Parasitology and Mycology, School of Public Health, Food Microbiology Research Center, Tehran University of Medical Sciences, Tehran, Iran.*; c *Department of Toxicology and Pharmacology, School of Pharmacy, Shahid Beheshti University of Medical Sciences,Tehran, Iran.*

**Keywords:** Wheat, UHPLC-MS/MS, Mycotoxins, Myco6in1^+^, Simultaneous Analysis.

## Abstract

This study focuses on optimization and validation of an Ultrahigh-performance liquid chromatography tandem mass spectrometry (UHPLC-MS/MS) method for simultaneous analysis of 11mycotoxins: Aflatoxins (B_1_, B_2_, G_1_, and G_2_), Ochratoxin A, Deoxynivalenol, Fumonisins (B_1_ and B_2_), Zearalenone, T-2, and HT-2toxin, in wheat matrix. Sample extraction and cleanup procedure is based on a single extraction step using acetonitrile/water/acetic acid mixture (79.5/20/0.5 v/v/v) and rapid clean-up of samples were performed with the Myco6in1^+^ Immunoaffinity column. Electrospray ionization at positive mode was operated to the simultaneously analysis of selected mycotoxins in a single run time of 15 min. Multiple Reaction Monitoring (MRM) mode was selected for quantification and detection of the mycotoxins. The analysis method was validated for selected mycotoxins at different spike levels (2-150 ngg^-1^ for AFs, T-2, OTA; 20-1500 ngg^-1^ for ZER, HT-2 toxin; and 100-1500 ngg^-1^ for DON and FB_1_+B_2_) in wheat. Calibration curves were plotted based on the area of peak analyte in spike samples. Limits of detection (LOD) ranged from 0.7 to 33.3 ngg^-1^ and limits of quantification (LOQ) ranged from 2 to 100 ngg^-1^. Recovery values were between 70 and 120% for all the mycotoxins, except for AFG_2 _(72-123%) and T-2 toxin (77-122%) with good repeatability. The recoveries and repeatabilities were in accordance with the criteria determined by European Union (EU) Recommendation 519/2014.

## Introduction

Mycotoxins are low-molecular-weight secondary metabolites of filamentous fungi which can cause adversely affect health in humans and animals. Mycotoxins are hepatotoxic, mutagenic, carcinogenic, or estrogenic effects and impair the immune system (1, 2,). Different species of mycotoxins can contaminate a wide range of food crop (fruits, cereals and grains). Hence, in Within the European Union and other countries are set maximum tolerance levels (MTL) for mycotoxins in different food (1, 3). Also, regulations have been set for the control mycotoxins in food and feed in Iran (4). Therefore, sensitive, accurate and reliable analytical methods are necessary for the analysis of the mycotoxins in cereal and cereal-based. Many official methods are available for determination of mycotoxins, but they are single target analyte methods in food and feed (5, 1) Analysis of simultaneous mycotoxins is necessary, Due to the structure of the complex food crop and naturally contamination by different fungal species. 

The majority is classical methods based on HPLC technique with FLD or UV detectors (6, 7) and in some cases, mycotoxins analysis was performed based on GC with ECD or MSD (8, 9). These methods focus more on the analysis of single compounds. Also, due to the cost, time consuming and need of trained operators and the inability to analyze multi- mycotoxins, they are not very acceptable (5). In the present, the methods based on LC/MS technique were performed with atmospheric pressure chemical ionization (APCI) and or electrospray ionization (ESI) ion source for ionization. These methods were validated in both positive and negative ionization modes (10-13). The most common MS/MS systems with different analyzers such as time of flight (TOF), ion trap, and triple Quadrapoles are used for simultaneous determination mycotoxins (14-18).

The different sample preparation methods are available for determining mycotoxins residues in food and feed using LC/MS/MS. In study Sulyok *et al. *(13); Warth *et al*. (19); Shimshoni *et al*. (17) and Blandino *et al*.(20), the sample preparation techniques were based a double extraction with organic solvents(water, acetonitrile and methanol) without clean-up. Zhang, Wu, Lu. (16) reported sample preparation procedure based on QuEChERS method without clean-up step for simultaneous carbamate insecticides and mycotoxins. In this approach PSA sorbent was not applied for cleanup extract. Aberg, Solyakov and Bondesson (21) described an extraction method that was being divided into two parts; without cleaning (OTA, FB1 and FB2) and cleaned extract with MultiSep®226 column.

In this context, the present investigation is the first designed study for the simultaneous determination of major mycotoxins namely AFs, Ochratoxin A, Deoxynivalenol, Zearalenone, FBs (B1+B2), T-2 and HT-2 toxin, in wheat matrix from Iran using LCMS/MS and Myco6in1^+^ Immunoaffinity column clean-up methods.

## Experimental

*Chemicals and reagents*

All mycotoxins standards were purchased from Sigma–Aldrich (St. Louis, USA). Also, all regent and solvents (all LC grades) were purchased from Sigma Aldrich (St. Louis, USA). Ultrapure water was obtained successively from a Milli-Q system (18.2 MU cm1Direct-Q3 UV, Merck, Germany). The Myco6in1^+^ Immunoaffinity column was obtained from VICAM (Watertown, MA, USA). 

*Standard preparation*

Stock standard solutions of AFs (AFB_1_, AFB_2_, AFG_1_, AFG_2_), ZER, OTA, FB_1_ (each 200 **µ**g/mL), DON, FB_2,_ and HT-_2_TOXIN (each 100 **µ**g/mL) were prepared by dissolving powder standard mycotoxins in a suitable solvent. But the stock standard solution of T-2 toxin (1 mg/mL concentration) was prepared by accurately weighs an appropriate amount of standard and dissolves it with Acetonitrile. Afterword intermediate standard solution T-2 toxin was prepared in 100 **µ**g/mL concentration with the same solvent. Stock solution standards of each mycotoxin were constructed in acetonitrile except AFB_1_ (in toluene/acetonitrile), OTA (in toluene /acetic acid) and FBs (in acetonitrile/deionize water). For all of the toxins except (T-2, HT-2 toxin and DON), purity is checked by spectrophotometry. The stock solutions were diluted with methanol in order to obtain working mix standard solutions (1 **µ**g/mL for AFs, OTA and T-2 –10 **µ**g/mL concentration for ZER, FB_1_, FB_2_, DON, and HT-2) of mycotoxins. Working mix standard solution was used for spiking the blank samples for linearity, repeatability, and trueness studies. The stock and working standard solutions were kept in the freezer at -20 °C.

*Sample preparation*

Extraction and clean up procedure was performed according to Myco6in1+ single extraction with using shaker for cereal method (22, 23). 5 g of homogenized wheat flour sample was weighed into a falcon tube (50 mL). Then 20 mL of extraction solvent of Acetonitrile: water: acetic acid (79.5:20:0.5 v/v/v) was added and shaken for 60 minutes on an orbital shaker (model 260B, Burladingen, Germany). Subsequently, the samples were centrifuged for 2min at 5000 r.p.m. After centrifugation, 2 mL supernatant was transferred into a 15mL falcon tube and was evaporated under nitrogen to dryness. After evaporation, 10 mL of Phosphate buffer saline (PBS) was added to falcon and was shaken for 3 min with multi reax shaker (Heidolph, Germany). Then 10 mL of the re resuspended extract was passed completely through the Myco6in1^+^ IAC. After washing the Myco6in1^+^ IAC with 10 mL deionized water, the mycotoxins were eluted with 3 mL methanol (2×1.5 mL). The eluate was dried under a nitrogen stream at 50 ºC and reconstituted with 1 mL of mixture Acetonitrile: water+0.01% acetic acid (50:50) and vortex for 3 min. Finally, the extract was filtered through a 0.45 μm syringe filter** (**25 mm PTFE Membrane 0.45 μm) and injected into the UHPLC/MS/MS analysis.

*UHPLC–MS/MS equipment and parameters*

The UHPLC-MS/MS system was performed using with a Phenomenex Security Guard ULTRA 10 mm × 2.1 mm i.d. guard cartridge coupled with a Phenomenex Kinetex XB-C18 (100 mm × 2.1 mm i.d., 2.6 μm). UHPLC column on an Dionex® 3000 ultimate LC system (USA) with an upper pressure limit of 600 bar, equipped with a binary pump(HPG-3400SD), autosampler(ACC-3000T), a column oven (TCC-3000SD) and interfaced with an API 3200™ triple quadrupole (QqQ) mass spectrometer (AB Sciex, foster City, USA). The ionization source was electrospray ionization (ESI). Chromatographic separation was set at 40 **°C** with flow rate 0.3 mL/min^-1^. An elution of solvent (A) consisted of water+0.1%formic acid and eluent B of methanol+0.1% formic acid; both contained 10 mM ammonium formate were used as mobile phase. The Chromatographic elution was as follows: 0 min, 95% of solvent A + 5% of solvent B; 2 min, 60% A + 40% B; 10 min, 0% A + 100% B; 11.5 min, 0% A + 100% B; 12 min, 95% A + 5% B; 15 min, 95% A + 5% B. The total run time of the LC was 15 min with injection volumes 20 **µ**L.

Mycotoxins were recorded in the MRM mode in positive polarity, with two transitions per mycotoxins (1 quantifier, 1 qualifiers). The instrument parameters were: source temperature 400 °C, collision-activated dissociation gas (CAD) 10, nebulizer gas (GS1) 50 psi, auxiliary gas (GS2) 50 psi, Curtain gas (CUR) 20 psi, and ion spray voltage +4500V. To optimize the MS/MS parameters for each mycotoxins, the tests were operated by direct infusion of an individual standards solution of the 1 **µ**g/mL concentrate in solvent MeOH: H2O (50:50 v/v) containing 5 mM ammonium format+0.1% formic acid into the mass spectrometer by using an infusion pump at a flow rate of 10 **µ**L/min. Summarizes the parameters of the optimized MRM transitions Table 1.

*Method validation*

The performance of validation methods carried out in accordance with Commission Regulation European Union No 519/2014(24). The validation parameters were demonstrated in terms of linearity, repeatability and trueness, as well as limits of detection and quantification (LOD & LOQ). The coefficient of linearity was calculated using wheat flour samples that were spiked with each Mycotoxins at the following levels: 2, 5, 10, 20, 50, 100,150 ngg^-1^ for AFB_1_, AFB_2_, AFG_1_, AFG_2_, T-2, OTA; 20, 50, 100, 200,500,100, and 1500 ngg^-1^ for ZER , HT-2; and 100, 200, 500, 1000,1500 for DON and FB_1_+B_2_ .The accuracy(recovery) and intermediate precision (repeatability (relative standard deviation (%RSD)) of Myco6in1+ single extraction method were evaluated through recovery experiments by spiking mycotoxins to a blank wheat sample at three different levels (3,15,75 ngg^-1^ for AFB1, AFB2, AFG1, AFG2, T-2, OTA; 30,150,750 ngg^-1^ for ZER , HT-2 toxin; and 150, 750, 1200 ngg^-1^ for DON and FB_1_+B_2_), tree replicates at each level (n = 3) at tree days.

## Results and Discussion

*Optimization of UHPLC-MS/MS conditions*

MS/MS optimization carried out with full scan experiments each is selected mycotoxins with direct injection of individual standard at 1µg/mL (AFB_1_, AFB_2_, AFG_1_, AFG_2_, FB_1_, B_2_, DON), 2 µg/mL (OTA, T-2 and HT-_2_ toxin) and 5 µg/mL (ZER) in the positive mode. Investigations showed that all of mycotoxins mentioned are able to create parent and product ions in both positive and negative. For example, in previous studies, some researchers have optimized the negative mode for deoxynivalenol and Zearalenone (13; 25). But there are few researches that have been performed to optimize the positive ion polarity (15; 26; 12). In addition, a switch from positive to negative mode and opposite, due to the increase in run time, reduce the sensitivity of the measurement and analysis performing in the two run time (27). So according to the above reasons, MS/MS parameters were optimized for all mycotoxins in the positive ESI mode. In all the cases, Mycotoxins were detectable in the forms of [M+H]+, except for T-2 and HT-2 toxin were detected as ammonium adduct ion [M+NH_4_]+. 

**Figure 1:**
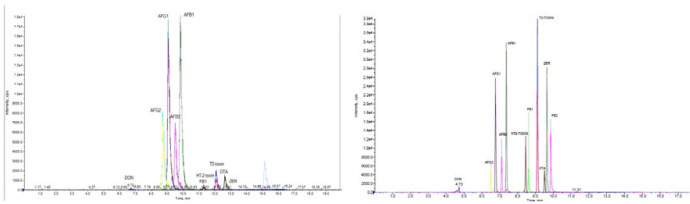
Sample Extract Ion Chromatogram column A (Chromolith performance, RP-18e 100-4.6mm) and Column B (kinetex , 2.6 μm XB-C18 100 mm × 3 mm i.d.)

Chromatographic separations of Mycoto-xins were carried out to determine the optimal conditions, using H_2_O/MeOH and H_2_O/ACN as the mobile phase under the gradient conditions. These two solvents (MeOH, ACN) are congruous with both reverse phase chromatography and MS (28). Anyway, most methods for the simultaneous mycotoxins analysis of methanol are used as the mobile phase (15, 26, 10, 29; 12). This can be due to poor solubility of methanol in C18 that will cause a stronger elution methanol. In some studies, also have been observed that the acetonitrile due to decreased ionization and sensitivity (28). In addition, for getting well the sensitivity, ammonium formate, or formic acid was added to the mobile phase. In order to better optimization of the elution phase and ionization conditions, formic acid and ammonium format was used. The results showed that Mycotoxins were successfully detected when formic acid was utilized in this way, and the sensitivity was improved. Selection of LC columns with the aim of obtaining better separation efficiency, two selective LC columns, namely (A) RP-18e 100-4.6mm (Merck, Darmstadt, Germany), Chromolith performance and (B) a XB-C18, 100 mm × 2.1 mm i.d., 2.6 μm, (Phenomenex, Macclesfield UK) were monitored for their separation efficiencies. The UHPLC-MS/MS chromatograms of Mycotoxins standards achieved with the two different columns under the similar operative conditions. The separation efficiency and sensitivity of column B (kinetex, XB-C18) was better than column A (Chromolith, RP-C18) (Figure 1). With the optimized conditions, the total run time was 15 min.

*Optimization of the extraction procedure*

In the present study, the Myco6in1+ single extraction procedure, as described by VICAM (22) was employed to extract intended mycotoxins. Currently, there are two multi-analyte methods for mycotoxins, a dilute-and-shoot and other a method based on multi-toxin imminoafinity column (IAC) (30). In this study, multi-toxin IAC method was used in combined with UHPLC-MS/MS. Myco6in1^+^ LC-MS/MS IAC is specific antibodies for 6 major mycotoxins (AFs, OTA, ZER, DON, FBs, T-2 and HT-2 TOXIN). The principle of the IAC is based on antibodies that entrap mycotoxins of interest. In Iran, this is the first study on the use of the Myco6in1+ single extraction method for the sample preparation step of selected mycotoxins in wheat flour before UHPLC analysis. In this extraction protocol the mycotoxins were extracted with20 mL acetonitrile: water containing acetic acid by the shaker and the clean-up was performed on to Myco6in1^+^ IAC. The results displayed that the solvent mixture acetonitrile/ water/ acetic acid (79.5:20:0.5 v/v/v) was the best compromise for the extraction of the selected mycotoxins from wheat flour. In this method, one Myco6in1^+^column is use for one sample to detect the multi-mycotoxins due to saving time and materials. In comparison with Solid Phase Extraction (SPE) and QuEChERS methodology, Myco6in1^+^ IAC is more specific for analyzing 6 major mycotoxins. Also, Myco6in1^+^ columns are compatible with photodiode array (PAD) and fluorescence (FL) detector. Myco6in1^+^ IAC column covers are all mycotoxins that have been authorized in Iran and the European Union. 

*Validation of the proposed method*

In the simultaneous mycotoxins analysis, Matrix effects are common problems when using LC-MS/MS. These matrix components have adverse impact on ionization of the target compounds and suppression or enhancement response compounds (1). Thereby, in this study was used a spike calibration curve to overcome the matrix effects. The linearity of the method was tested by spike samples at seven concentration levels; 2, 5, 10, 20, 50, 100,150 ngg^-1^ for AfB_1_, AfB_2_, AfG_1_, AfG_2_, T-2 TOXIN, OTA; 20, 50, 100, 200,500,1000 and 1500 ngg^-1^ for ZER , HT-2 TOXIN; and 100, 200, 500, 1000,1500 for DON and FB_1_+B_2_.with respect to the MTLs. The linearity studies were repeated on three different days. The calibration curve was achieved by plotting the peak area compound in the range 2-150 ngg^-1^ for AFB_1_, AFB_2_, OTA; 5-150 ngg^-1 ^for AFG_1_,AFG_2_, T2-toxin;50-1500 ngg^-1^ for ZER; 20-1500 ngg^-1^ for HT-2 toxin and 100- 1500 ngg^-1^ for DON,FB_1_,FB_2_. Which are presented in Table 2. 

Correlation coefficients (R^2^) were obtained for all the target mycotoxins in the range of 0.99-0.9999 for the seven point calibration curves. Detection limits and quantification limits were calculated in spiked blank samples, and they were determined as the lowest amount of each mycotoxins with a signal-to-noise ratio(S/N) of 3/1 and 10/1, respective. The ranges of LOQs and LODs were 2-100 ngg^-1^ 0.7-33.3 ngg^-1^ for all of the selected mycotoxins in wheat flour samples. AFB_1_, AFB_2_ and OTA were shown the lowest level of LOQs (2 ngg^-1^) and LODs (0.7 ngg-1). The limits of quantitation (LOQs) for all of the intended Mycotoxins are lower than their Maximum Tolerated Limits (MTLs) set by European Union (EU) and Institute of Standard and Industrial Research of Iran (3, 4) in cereal, particularly in the wheat. For all the analyte, repeatability (RSDr) was equal or lower than 20%, except for HT-2 toxin and FB2, which show some values higher than 20%.but it was congruous with the EU regulation (24). The recovery of the extraction step for all of target mycotoxins were spiked on blank samples at three different concentrations. The mean recoveries varied from 72 to 123%, and the range of repeatability (RSDr) was 0.6% to 24.2%, respectively. According to the Commission Regulation European Union No 519/2014 document (24), in most of the cases the RSDr should be lower than 20%, except for Zeralenon, fumonisins, T-2 and HT-2 toxin (for example HT-2 toxin: RSDr ≤ 25% and Recovery 60-130% of spike level > 250 mg-1) with due attention to spike level.

Therefore, good recoveries from wheat samples were achieved throughout the developed method, indicating the suitability of the proposed extraction procedure for the simultaneous extraction of selected mycotoxins from wheat samples. The recoveries and repeatabilities were in accordance with the criteria determined by the Commission of the European Communities (24). The results are shown in Table 3. 

Our results are in accordance with recent findings by Frenich *et al* (12), Spanjer *et al*. (15). Frenich *et al* reported the range recovery between 70.0%- 104.8% with RSD lower than 25%.

**Table 1:** MS/MS parameters for the detection of 11 mycotoxins in the positive ESI mode (MRM program)

**Precursor Ion**	**product ions**	**R**_t_^b ^ **(min)**	**NAME**	**DP** ^c^	**EP** ^d^	**CEP** ^e^	**CE** ^f^	**CXP** ^g^
**297.1**	249.2	4.7	DON^a^	31	7.5	22	19	12
**297.1**	203.3	4.7	DON	31	7.5	22	25	4
**313.0**	241.2	7.3	AFB_1_^a^	56	7	24	33	4
**313.0**	213.2	7.3	AFB_1_	56	7	24	49	4
**315.1**	259.2	7.1	AFB_2_^a^	66	3.5	26	35	4
**315.1**	287.2	7.1	AFB_2_	66	3.5	26	29	14
**329.0**	200.3	6.7	AFG_1_^a^	41	12	24	45	4
**329.0**	243.2	6.7	AFG_1_	41	12	24	25	4
**331.1**	245.3	6.5	AFG_2_^a^	61	6	28	31	4
**331.1**	201.2	6.5	AFG_2_	61	6	28	45	4
**722.3**	334.4	8.6	FB_1_^a^	71	7	42	49	16
**722.3**	316.4	8.6	FB_1_	71	7	42	49	14
**706.2**	336.2	9.8	FB_2_^a^	66	7.5	48	51	14
**706.2**	318.5	9.8	FB_2_	66	7.5	48	51	14
**442.2**	215.3	8.4	HT-2 toxin^a^	21	4	30	23	4
**442.2**	263.3	8.4	HT-2 toxin	21	4	30	29	12
**404.0**	102.2	9.5	OTA^a^	26	6	26	91	2
**404.0**	239.2	9.5	OTA	26	6	26	31	4
**484.3**	215.2	9.1	T2-TOXIN^a^	21	6.5	32	31	4
**484.3**	245.3	9.1	T2-TOXIN	21	6.5	32	27	4
**319.1**	187.2	9.6	ZER^a^	26	5	24	23	4
**319.1**	185.2	9.6	ZER	26	5	24	33	4

AFB1: Aflatoxin B_1_;AFB_2_ Aflatoxin B_2_;AFG_1_ Aflatoxin G_1_;AFG_2_ Aflatoxin G_2_; CE: collision energies; DON: deoxynivalenol; DP:

Declustering ;potential; EP: entrance potential; ESI: electrospray ionization;FB_1 _fomonizine B_1_;FB_2_ fomonizineB2; MS:mass spectrometry;

OTA: ochratoxin A; R_t_: retention time; ZER:zearalenone.

^a^Quantitation.

^b^Retention time.

^c^Declustering potential.

^d^Entrance potential.

^e ^Collision Cell Entrance Potential

^f^Collision energies.

^g ^Collision Cell Exit Potential

**Table 2:** Results obtained from spike calibration curve

Analyte	calibration curve								MTL in unprocessed cereals (ng g^-1^)	
	***r2***	***a***	***b***	**Cal. Range** **(ng g** ^-1^ **)**	**LOQ** **(ng g** ^-1^ **)**	***LOD*** ***(ng g*** ^-1^ ***)***	**Accuracy (%)**	**(%)** **range of RSDr**	**ISIRI**	**EU**
**AFB1**	0.9998	874.6	-404.5	2-150	2	0.7	93.1	5.2-21.1	5	2
**AFB2**	0.9999	407.2	-572.5	2-150	2	0.7	102.6	2-17.8	15^1^	4
**AFG1**	0.9964	492.5	-1915.2	5-150	5	1.7	103.5	0.8-18.9		
**AFG2**	0.9991	107.9	458.9	5-150	5	1.7	94.8	3-14.3		
**OTA**	0.9996	149.6	-23.6	2-150	2	0.7	102.1	2-18.1	5	5
**DON**	0.9973	2.3	29.3	100-1500	100	33.3	105.0	1.7-20.9	1000	1250
**FB1**	0.9955	8.6	-637.3	100-1500	100	33.3	105.0	3.4-14.3	1000^2^	1000
**FB2**	0.9998	5.1	-281.8	100-1500	100	33.3	100.0	9.3-26		
**ZER**	0.9992	41.9	-457.3	50-1500	50	16.7	101.6	2.7-17.9	200	100
**T-2 TOXIN**	0.9994	107.8	46.2	5-150	5	1.7	97.6	1.1-13.4	..	..
**HT-2 TOXIN**	0.9981	24.4	-202.6	20-1500	20	6.7	105.4	4.2-21.4	..	…

^1^Sum of AF_S_

^2^Sum of FB_1_ + FB_2_

## Conclusion

In this study, we exhibited a rapid and sensitive screening method for analyzing 11 mycotoxins, including AFs, OTA, ZER, DON, FB_1_ + B_2_, T-2, and HT-2 toxin. The method is selective and specific for multi analysis of mycotoxins, including a single extraction step with a cleanup step based on Myco6in1^+^ Immunoaffinity column.

The Myco6in^1^ IAC had good recovery of for all mycotoxins. In addition, the clean-up procedure was simpler and safer for MS/MS system than the other sample preparation methods.
